# Single-Center Experiences of Preoperative Juvenile Nasal Angiofibroma Embolization With Gelfoam, Reducing Financial Burden on Patients in Developing Country

**DOI:** 10.7759/cureus.18378

**Published:** 2021-09-29

**Authors:** Junaid Iqbal, Kamran Fazal, Sadia Rashid, Shahmeer Khan, Jehanzeb Shahid, Danial Khalid

**Affiliations:** 1 Radiology, Aga Khan University Hospital, Karachi, PAK; 2 Radiology, Patel Hospital, Karachi, PAK; 3 Radiology, Dr Ziauddin University Hospital, Karachi, PAK

**Keywords:** vascular tumor, endovascular, gelfoam, catheter, embolization, juvenile nasal angiofibroma (jna)

## Abstract

Introduction

Juvenile nasal angiofibroma (JNA) is a highly vascular tumor of the nasopharynx. Endovascular embolization followed by surgery is the treatment of choice. This study aimed to determine that single catheter technique with Gelfoam is an effective and safe technique for embolization to reduce the financial burden on patients in a developing country.

Materials and methods

We retrospectively reviewed the imaging, surgical, and histopathological records of 108 patients who underwent preoperative endovascular tumor embolization followed by surgical resection between March 2017 and March 2021.

Results

After embolization no major complication was observed in any patient. Complete devascularization of tumor was done in 87.8%. Intraoperative blood loss resulting in transfusion was almost the same as with other embolization techniques.

Conclusion

Single catheter with Gelfoam is a cost-effective and safe technique for JNA embolization.

## Introduction

Juvenile nasal angiofibroma (JNA) is a rare but most common nasopharyngeal benign tumor that occurs exclusively in adolescent males and it accounts for 0.5% of all head and neck tumors [1-3]. Patients are usually asymptomatic when tumors are small but when the tumor is enlarged nasal obstruction and epistaxis are the main presenting features [1,2]. JNAs are locally aggressive benign tumors centered on the sphenopalatine foramen that may show extension into the nasal cavity, maxillary sinus, infratemporal fossa, and middle cranial fossa [4,5]. JNAs are highly vascular tumors receiving blood supply mainly from the branches of the ipsilateral external carotid artery. Large tumors receive blood supply from the branches of the contralateral external carotid artery and rarely from the internal carotid artery [6]. Because of being hypervascular, there is a high risk of massive blood loss during surgery. Therefore, preoperative endovascular embolization is the most utilized technique to reduce morbidity and mortality secondary to perioperative blood loss [7]. There are various challenges during the procedure like the superselection of small tortuous arterial feeders, which may lead to vasospasm or arterial dissection. In addition, neurological complications like stroke are also possible from accidental migration of embolizing agents into the brain through extracranial to intracranial anastomoses [8-10]. Various agents are widely available for embolization, and the selection of embolizing agents depends upon the operator preference and cost-effectiveness [11].

## Materials and methods

After getting approval from the Departmental Research Committee, we retrospectively reviewed the imaging, surgical, and histopathological records of 108 patients who underwent preoperative endovascular tumor embolization followed by surgical resection between March 2017 and March 2021. The procedures were carried out using a Seimens Artis one monoplane (Germany). Preoperative contrast-enhanced CT scans were obtained for diagnosis, size, extension, and location. Before the procedure, informed consent was taken, and the interventionist explained the procedure and the possible complications. The risks and complications of the procedure include pain, hematoma at the puncture site, post-embolization damage to surrounding structures like nerves and blood vessels, and accidental nontarget embolization resulting in potentially irreversible damage to vital structures or stroke. Initially, we performed a diagnostic angiogram to determine the arterial blood supply to the tumor and to evaluate for dangerous collaterals to the intracranial circulation. The most common pathways of external to internal carotid anastomoses are in the orbital region via the ophthalmic artery. The most common feeding artery to nasal angiofibroma was the internal maxillary artery and its branches, i.e., the sphenopalatine and descending palatine arteries. Procedures were performed under light sedation without general anesthesia. Percutaneous femoral artery access was obtained by Seldinger technique. A 4 French vascular sheath was placed in the femoral artery. Diagnostic cerebral angiography was performed with selective catheterization of the internal and external carotid arteries bilaterally using a 4 French diagnostic catheter over a guidewire (0.035 inch). A catheter was then placed into the origin of the external carotid artery on both sides to see the feeding artery to the tumor. A 4 French catheter was advanced over the guidewire without using a microcatheter. Angiography was performed again to determine the branch of an artery supplying the tumor and to identify accessory or aberrant arterial supply. Selective embolization of the artery was then performed using Gelfoam slurry only. If there was tumor supply from the contralateral external carotid artery, this was also embolized. Intermittent angiograms were performed, and embolization continued till maximum tumor devascularization was done or if there was reflux of Gelfoam slurry because it would be unsafe for the patients. The distal internal maxillary and distal facial arteries were commonly embolized. At the end of embolization, bilateral external carotid artery arteriogram was performed to see tumor devascularization. All patients underwent surgical resection of the tumor within 48 hours following endovascular embolization except in three patients in whom surgery was delayed and we performed re-embolization afterward. Postoperative notes and hospital records were also reviewed for documentation of operative time, intraoperative blood loss, transfusion requirements during surgery, and postoperative hospital stay. Data analysis was performed using SPSS version 21 (IBM Corp, Armonk, NY), and frequency and percentages were computed to express the findings. The non-utilization of microcatheter and polyvinyl alcohol (PVA) particles in our study resulted in significant reduction in cost of the procedure.

## Results

All the 108 nasopharyngeal angiofibroma cases in which we performed endovascular embolization had arterial feeders from the internal maxillary artery branches (sphenopalatine and descending palatine arteries). In 68 patients (62.9%), tumors were getting supply from the bilateral internal maxillary artery, in 24 patients (22.8%) only from the right, and in 16 patients (14.8%) only from the left internal maxillary artery. In 14 patients (12.9%) tumors were also getting feeders from ascending pharyngeal artery. In nine patients (8.3%), tumors were also getting supply from branches of the internal carotid artery (Table 1). In those patients we modified our conventional technique and used microcatheter with onyx for embolization under general anesthesia, so those were not included in our study.

**Table 1:** Blood supply to juvenile nasal angiofibromas in this study, showing majority of patients getting blood supply from branches of bilateral external carotid artery. Data presented as n (%).

Bilateral internal maxillary artery	Right internal maxillary artery	Left internal maxillary artery	Ascending pharyngeal artery (any side)	Branches of internal carotid artery (any side)
62.9	22.8	14.8	12.9	8.3

Following embolization, seven patients out of 99 patients (7.0%) experienced severe pain, which required sedation, four patients (4.0%) experienced bradycardia for few minutes; however, it reverted spontaneously with an injection of analgesics. No patient experienced accidental non-targeted embolization of intracranial circulation (Table 2). According to the surgical record, surgeons reported almost complete devascularization of tumor in 87 patients (87.8%) while partial devascularization was achieved in 11 patients (11.1%) (Table 3). The average duration of surgery was 155 minutes (80-185 minutes). The average blood transfusion requirement during surgery was 190 ml (50-400 ml), and these are almost the same as with other embolization techniques. No procedural or surgical complications were encountered in any of the cases in our study. No neurological deficit or stroke was observed after the embolization.

**Table 2:** Post-procedure complications in this study, showing that no serious complications were observed in our adopted technique of embolization. Data presented as n (%).

Severe pain	Bradycardia	Accidental intracranial embolization
7	4	0

**Table 3:** Surgical outcome: complete devascularization of tumor was reported by surgeons in a majority of patients. Data presented as n (%).

Complete devascularization	Partial devascularization
87.8	11.1

## Discussion

JNA is a rare nasopharyngeal benign tumor of adolescent males and comprises around 0.5% of head and neck tumors [3]. They are locally aggressive arising from sphenopalatine foramen showing extension and invasion into the adjacent structures and even intracranially [4,5]. Nasopharyngeal angiofibroma receives a rich blood supply commonly from the branches of the external carotid artery, predominantly the internal maxillary artery and its branches (sphenopalatine and descending palatine). Larger tumors get blood supply from the bilateral external carotid artery and even feeders from the internal carotid artery [7]. The abnormal tumor vessels of JNAs only containing endothelial layer without muscular layer, therefore, increase the risk of bleeding either spontaneously resulting in recurrent epistaxis or may cause life-threatening and uncontrolled bleeding during surgical removal of a tumor [3].

To avoid perioperative bleeding, preoperative endovascular embolization of nasal angiofibroma is the preferred treatment of choice [7,12-14]. Preoperative embolization with particulate or liquid embolic agent has significantly reduced perioperative blood loss and operative time [7]. Selective catheter placement in small and tortuous branches of an artery may cause difficulty during the preoperative endovascular embolization [8-10], and this sometimes becomes more difficult when performed without a microcatheter for cost-saving. In our patients who were included in this study, we used a 4 French catheter, which has a smaller caliber as compared to a 5 French one, and this can easily engage the internal maxillary artery and even its branches. Additionally, the presence of dangerous extracranial-to-intracranial anastomoses and the inadvertent reflux of particles into the intracranial circulation may increase the risk of neurologic complications [10]. Rarely JNAs are also supplied from the branches of the internal carotid artery and develop external carotid-internal carotid anastomoses. During an initial diagnostic angiogram, JNAs that show external carotid-internal carotid anastomoses are recommended to be embolized by some modified techniques like using microcatheter, preferring liquid embolic agent over particulate agents, or adopting balloon-assisted embolization for the safety of the procedure. Otherwise, there is a risk of intracranial migration of particles resulting in serious neurological complications like stroke and vision loss [15-21].

Various embolic agents including Gelfoam, PVA particles, and onyx are available for embolization of JNAs. Each material has its advantages and disadvantages. Gelfoam is cost-effective, and the size can be controlled by an operator; as Gelfoam is a temporary embolic agent, postembolization recanalization occurs early (48 hours) as opposed to permanent embolic agents like PVA particles and onyx but the cost of a permanent embolic agent is very high as compared to Gelfoam [22-24]. In a study conducted by Seok Hahn et al. [22], they compared the clinical outcomes of PVA and Gelfoam in bronchial artery embolization. They found that there was no significant difference in the results of Gelfoam and PVA groups immediately after the procedure and after one month. However, PVA had better results than Gelfoam after 12 months.

The limitation of this study is that we could not perform this conventional technique of embolization in those patients in whom tumors get even minor supply from branches of the internal carotid artery and so those were not included in our study.

## Conclusions

Preoperative embolization of JNA by using Gelfoam and a single-catheter technique appeared to be a safe and effective devascularization method to reduce perioperative blood loss and operating time. By this technique, we significantly reduced the financial burden on patients as we are living in a third-world country with financial constraints. By avoiding microcatheter and permanent embolic agents we reduced the cost of the procedure up to 80% with good perioperative devascularization without significant complications. However, the selection of patients for a single-catheter technique for embolization during initial diagnostic angiogram is very important to avoid serious neurological complications. 
